# Design methodology of portable upper limb exoskeletons for people with strokes

**DOI:** 10.3389/fnins.2023.1128332

**Published:** 2023-03-16

**Authors:** Yongkun Zhao, Haijun Wu, Mingquan Zhang, Juzheng Mao, Masahiro Todoh

**Affiliations:** ^1^Division of Human Mechanical Systems and Design, Graduate School of Engineering, Hokkaido University, Sapporo, Japan; ^2^Department of Bioengineering, Faculty of Engineering, Imperial College London, London, United Kingdom; ^3^Division of Mechanical and Aerospace Engineering, Faculty of Engineering, Hokkaido University, Sapporo, Japan; ^4^State Key Laboratory of Bioelectronics, Jiangsu Provincial Key Laboratory of Remote Measurement and Control, School of Instrument Science and Engineering, Southeast University, Nanjing, China

**Keywords:** portability, upper limb, exoskeleton, stroke, rehabilitation

## 1. Background

**Figure 1:**
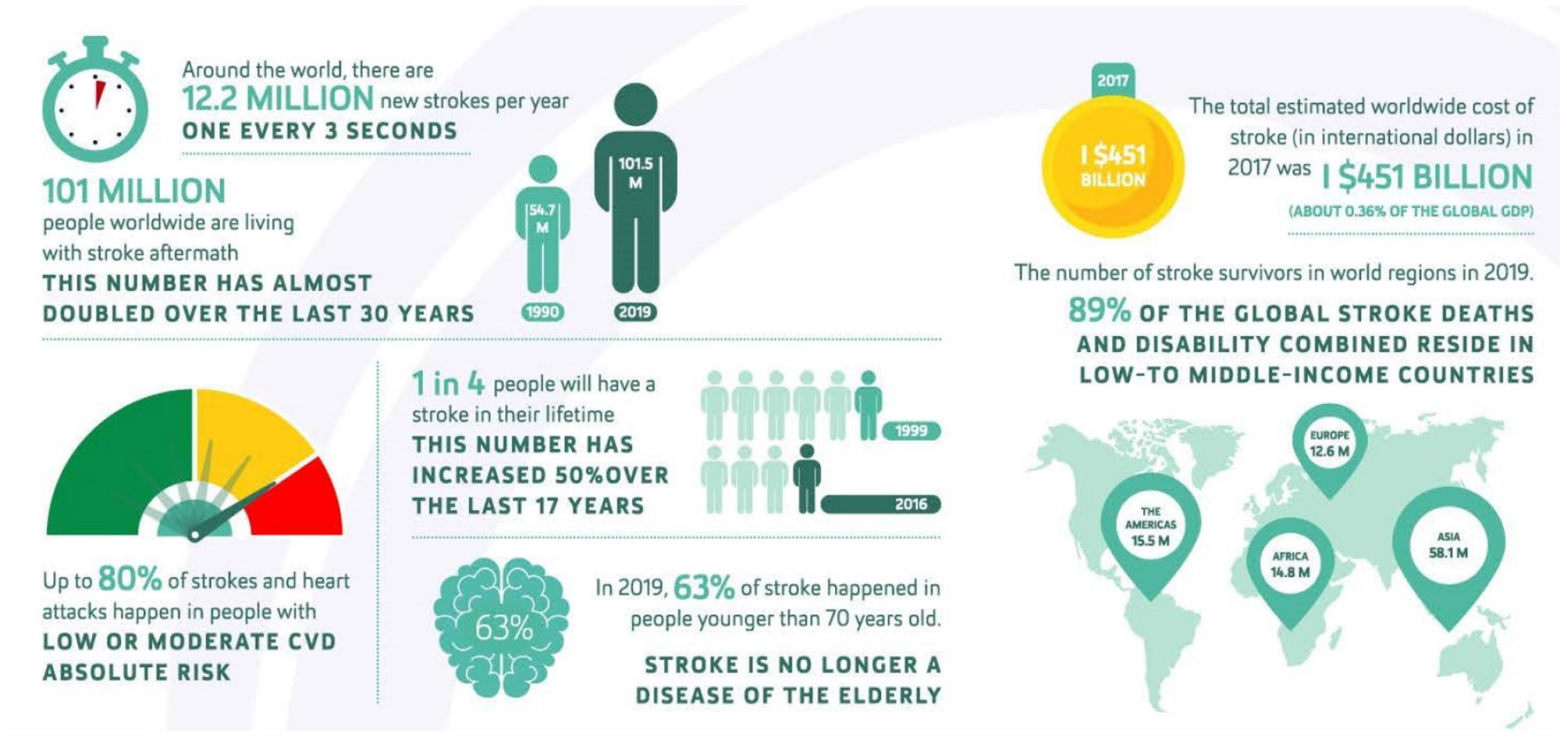
Stroke infographic from World Stroke Organization (WSO): global stroke fact sheet 2022 (Feigin et al., 2022).

According to the report released by the World Stroke Organization (WSO) in 2022, a new stroke occurs on average every 3 seconds, with 12.2 million new cases of stroke reported year globally (Feigin et al., 2022). The aftereffects of a stroke are now experienced by 101 million individuals globally. This number has almost doubled in the last 17 years. In 2016, one in four people had a stroke in their lifetime, compared to one in six in 1999. Nowadays, there are so many stroke cases that it has become a substantial economic burden for society. The total global expenditures on treatment and related research rose to United States Dollars (USD) 145.1 billion in 2017, and this amount was around 0.36% of the world gross domestic product (GDP) for that year. This information is visualized in Figure 1.

In order to treat stroke patients, medical centers are required to provide specialized rehabilitation training. Traditional rehabilitation training methods with human intervention have proven to be effective for stroke patients. However, the high cost and lack of sufficient experienced therapists result in many patients not receiving adequate rehabilitation training (Clarke et al., 2015). Therefore, patients need to use an automated device for rehabilitation instead of manual training. Since then, a lot of exoskeletons have been developed to provide post-stroke patients with sufficient rehabilitation services (Norouzi-Gheidari et al., 2012). All of them can be mainly classified into two types based on the mechanical structure: ground-based exoskeleton and body-based exoskeleton (Manna and Dubey, 2018). Portable exoskeletons are more likely to be employed extensively in the rehabilitation process of stroke patients compared to bulky ground-based exoskeletons. This paper provides an overview of the recent technologies of portable upper limb exoskeletons and suggests potential advancements in the near future.

## 2. Portable upper limb rehabilitation exoskeleton

Exoskeleton robots are well known to be highly nonlinear mechatronic systems. Researchers from many different fields have shown a great deal of interest in upper limb rehabilitation exoskeletons, which resemble the human arm's anatomical structure and work in parallel with the affected limb. Examples include Bones (Klein et al., 2010), RUPERT (Huang et al., 2015), CAREX (Mao et al., 2014), ARMin (Mihelj et al., 2007), and IntelliArm (Ren et al., 2012). The effectiveness and portability of a portable upper limb exoskeleton for the rehabilitation of stroke patients are significantly impacted by its physical properties. An exoskeleton's portability is determined by its actuation system's weight and material properties. Choosing the right drive system and operation mode will ensure that the exoskeleton generates constant and consistent torque, thus ensuring its therapeutic effectiveness. Therefore, this section summarizes the development of portable upper limb rehabilitation exoskeletons in recent years from three aspects: physical properties, actuation system, and operation modes.

### 2.1. Physical properties

**Figure 2:**
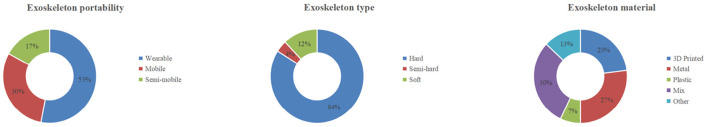
The distribution of physical properties of the upper exoskeletons in terms of portability, exoskeleton type, and structural material (Vélez-Guerrero et al., 2021).

The physical properties of portable exoskeletons determine their portability and effectiveness in clinical application. Figure 2 shows the overall distribution of physical properties of portable exoskeletons summarized in the key assessment criteria (Vélez-Guerrero et al., 2021).

#### 2.1.1. Exoskeleton portability

In terms of portability, portable exoskeletons can be divided into three categories: wearable exoskeletons, where all structures are entirely contained within the mechanical structure parallel to the patient's limb; mobile exoskeletons, where the motor part is situated on the movable platform; and semi-mobile exoskeletons, which require some additional support structures. Wearable exoskeletons are discussed in around 53% of papers in the literature on portable upper limb exoskeletons for rehabilitation (Vélez-Guerrero et al., 2021). There were certain cable-operated exoskeletons in this sector that had the most portability because of their flexible and lightweight main construction. For example, Varghese et al. (2020) emphasize the lightweight of the gadget and their invention weighs around 950 g. Thirty percent of the literature was on mobile robot exoskeletons. Despite being portable and operating without a fixed framework, they take longer to install because of their complexity, size, or weight. According to a case study done by Seeland et al. (2017), it has been demonstrated that the exoskeleton in this article performs well. However, due to more than 20 kg of weight, it cannot be used in other situations besides professional training in hospitals. About 17% of the exoskeletons reporting semi-mobile exoskeletons were found. These exoskeletons require additional support structures, which limits the portability of exoskeletons in different environments, and limits the versatility of this kind of exoskeletons.

#### 2.1.2. Exoskeleton type

In terms of the degree of softness and hardness of the materials used for the portable exoskeleton, the exoskeleton can be classified into three categories: hard exoskeleton, soft exoskeleton, and semi-hard exoskeleton. Hard exoskeletons are the most common type of exoskeleton, with 84% of the literature devoted to them. A rigid exoskeleton is one whose structure bears all of the user's afflicted limb's weight and whose structure is, in most situations, considerably resistant to the strain or deformation caused by external forces. Hard exoskeletons can also be quite compact and provide some mechanical flexibility (Sangha et al., 2016). Twelve percent of the research literature is concerned with further advancements in flexible, soft exoskeletons. The user's unrestricted natural motion would be aided by the employment of a soft, flexible gadget (Samper-Escudero et al., 2020). Finally, 4% of the literature reports the addition of some soft materials to certain structures of hard exoskeletons. For example, Shamroukh et al. (2017) demonstrated an effective passive orthosis design featuring a properly tensioned viscoelastic band for a tight fit, where torque is directed to the wrist to provide the maximum mechanical effect.

#### 2.1.3. Exoskeleton material

The materials used to make the exoskeleton are also very important. Thirty percent of the articles about the structural materials employed in exoskeletons mention the presence of two or more materials. The most typical combination involves adding plastic components to metal constructions to enhance physical strength and minimize the weight of the exoskeleton. Structures made of different metals are also frequent, 27%, giving the structure stiffness. For instance, Rosales Luengas et al. (2018) consider aluminum alloy 6061 T6 to be the best material after extensive testing. The usage of completely printed structures, such as Acrylonitrile Butadiene Styrene (ABS), Polylactic Acid (PLA), or nylon plastic components, has also increased significantly with the introduction of additive manufacturing and 3D printers, accounting for 23% of the literature. For instance, the system designed by Al Bakri et al. (2018) verifies that the plastic material can work properly under numerous weights on the mechanical structure. The United States Food and Drug Administration's regulatory guidelines for use in medical devices were met by the selection of Taulman nylon 680 and polyethylene terephthalate glycol modified (PETG) plastic, both of which have acceptable mechanical qualities and are reasonably priced. In addition, only a tiny percentage of study reports, 13% of the review literature, use different types of soft or semi-soft materials.

### 2.2. Actuation system

**Figure 3:**
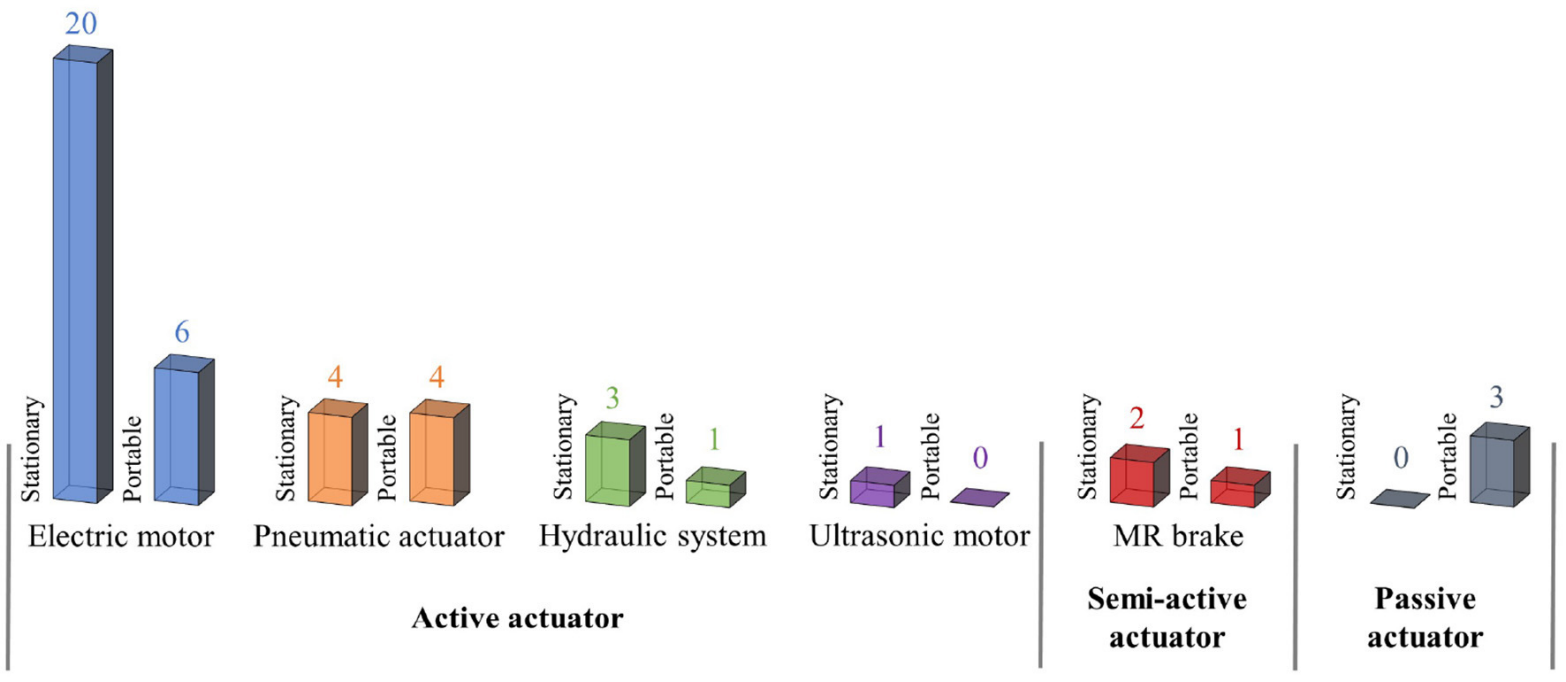
Statistics of actuators used for stationary and portable upper limb exoskeleton (Manna and Dubey, 2018).

The actuation system is the most important component in developing a portable exoskeleton as its weight strongly influences how portable the exoskeleton is. Therefore, choosing an actuator with a high power-to-weight ratio and the ability to create high torque with accurate movement is essential. In the market, several actuators have been used for the upper limb exoskeleton, and their statistics are shown in Figure 3. The most efficient actuators among them are electric motors, hydraulic motors, and pneumatic motors. Here, the benefits and drawbacks of adopting them for exoskeletons are discussed in detail.

The active actuator can generate a wide range of motions at varying speeds and torques. The traditional active actuators that are frequently employed in exoskeleton design include electric motors, pneumatic systems, and hydraulic systems. The semi-active actuator controls the joint stiffness according to the task requirement. The joint support is provided by passive actuators. It is based on passive components like springs or rubber belts that use their elastic qualities to produce force without requiring any energy.

#### 2.2.1. Electric motor

The motor actuator unit for the active exoskeleton is typically a motor with one degree of rotational flexibility. The brushed or brushless DC motor with the features of easy control and large power bandwidth is often chosen in the rehabilitation exoskeleton. These DC motors can be easily powered by the DC power supply. Additionally, because brushless motors operate in multiple phases, additional electronic components must be added to the control circuit. These additional electronic components are not required in the circuit for brush motors, so selecting a brushed motor can simplify the exoskeleton control circuit. Motors directly mounted on the exoskeleton structure are usually small motors, which are difficult to provide sufficient torque for the active mode. For example, the DC motor used by Ragonesi et al. (2011) is a FaulHaber brushed DC motor type 2342 S 012 CR with a 134:1 gear head. The motor is powered using an Advanced Motion Control 12A8 amplifier. It has a rated power of just 24 W, a diameter of around 23 mm, and a length of about 42 mm.

If the electric motor is able to generate enough torque to drive the exoskeleton at rated speed and that it is also able to perform active treatment under a variety of stresses, a rather large motor must be required to support the weight of the human arm. However, a big DC motor positioned at an exoskeleton joint may result in a number of dynamic problems. These problems will only become worse since an exoskeleton system may require three or more motors to power exoskeleton movement (Dragusanu et al., 2022). Since the motor at the shoulder joint must support the weight of the entire arm, including the motor of the mechanical structure and other pertinent components, the exoskeleton system as a whole will have significant inertia problems in this situation. A common way to reduce the exoskeleton system inertia is to move the motor position to a distance and transfer the force through a rigid linkage (Tondu and Lopez, 2000) or a cables system. In the exoskeleton driven by cables, although its range of motion is large (Brown et al., 2003; Landkammer et al., 2014; Manna and Dubey, 2018; Dragusanu et al., 2022), because cables can only provide tension, two cables and two actuators are needed to create two-way motion for the joints. In this case, the portability of the exoskeleton will be limited by the bulky mobile platform. For example, in NEUROExos (Cempini et al., 2013), carried on a dedicated adjustable appendix of the moveable stand support, the motor unit is composed of a DC servomotor (Maxon EC motor EC60, 400 W), a Harmonic Drive reduction stage (Harmonic Drive CPL 17A 080 2) with a reduction ratio of 80, a grooved pulley which the steel cables wrap around.

#### 2.2.2. Hydraulic motor

Even though among all exoskeleton types, the joints of hydraulically driven exoskeletons have the highest torque to weight ratio (Brown et al., 2003), they are not appropriate for portable systems due to the huge space have to be reserved for traditional hydraulic systems, as well as other significant difficulties including fluid leakage and non-linear drives (Lang et al., 2022). However, there are still several examples of hydraulic motors that can be adopted for exoskeleton such as hydraulic bilateral servo actuators (HBSA) (Umemura et al., 2009) and flexible fluid actuators (Stienen et al., 2008). For hydraulic bilateral servo actuators, the electric motor combined with a lead screw is used to pressurize the fluid. The hydraulic cylinder has the advantage of low transmission loss because the motor is very close to the hydraulic cylinder, but a significant drawback of this type of actuator is that one actuator can only support a single joint movement. All hydraulic cylinders of the exoskeleton can be powered by a single reservoir and a single pump. A modular fluid actuator called the Flexible Fluid Actuator has been used for elbow joints. It comprises flexible reinforced bellows that will expand under pressure. It allows for the rotational movement of the joint if it is attached between two connecting rods. The actuator is light and portable and is operated by a tiny hydraulic pump and a small portable reservoir.

#### 2.2.3. Pneumatic motor

Similar to hydraulic actuators in operation, pneumatic actuators are distinguished by the fact that they offer proportional movement in both directions using compressed air as opposed to hydraulic fluid. The power-to-weight ratio of pneumatic actuators is also high. The cylinder and the artificial muscle (Tondu and Lopez, 2000) are the two components that make up pneumatic actuators, which are now established products on the market (Kalita et al., 2022). The key advantage of artificial muscle is that, in comparison to other active actuators now on the market, it delivers a better torque to weight ratio. Additionally, compared to electric motors, it has a lower impedance. When the artificial muscle is pressurized by compressed air, it exhibits behavior similar to that of human muscle by expanding and contracting in two layers of woven nylon that are knitted together. Smoothness, lightness, and obedience are issues that this kind of actuator solves. Because of this, a pneumatic muscle-driven exoskeleton is often referred to as a soft robot. The exoskeleton is made more ergonomic by creating natural compliance in the construction. However, a number of issues still exist with the artificial muscle, including poor bandwidth, non-linear properties, unidirectional operation, and big bulk. It is challenging to assemble with other components in a tiny, cramped space because of its bigger size. A pair of pneumatic muscles are necessary to produce bi-directional joint movement because it can only move in one direction. The use of this actuator is challenging for joints that have several degrees of freedom, such as the wrist and shoulder joints.

#### 2.2.4. Other types of actuators

In addition to electromagnetic, pneumatic, and hydraulic actuators, other actuation concepts based on piezoelectricity, shape memory alloys, electroactive polymers, metal hydrides, and polymeric gels also exist. Among them, the electroactive polymers' properties allow for the possibility of some application in the future. The electroactive polymer is a polymer that can change its size or shape when voltage is applied. This concept of actuation is most comparable to the McKibben muscle. Because of this property, electrically active polymers are often referred to as artificial muscles. When high voltage is applied, the shape will shrink. Unfortunately, they can only contract. In addition, only a small displacement can be achieved (Kovacs et al., 2007). This kind of actuator may be of great significance to the development of flexible exoskeletons in the future because it has no noise problem of traditional pneumatic muscles and can increase the invisibility of the exoskeleton.

### 2.3. Operation modes

**Figure 4:**
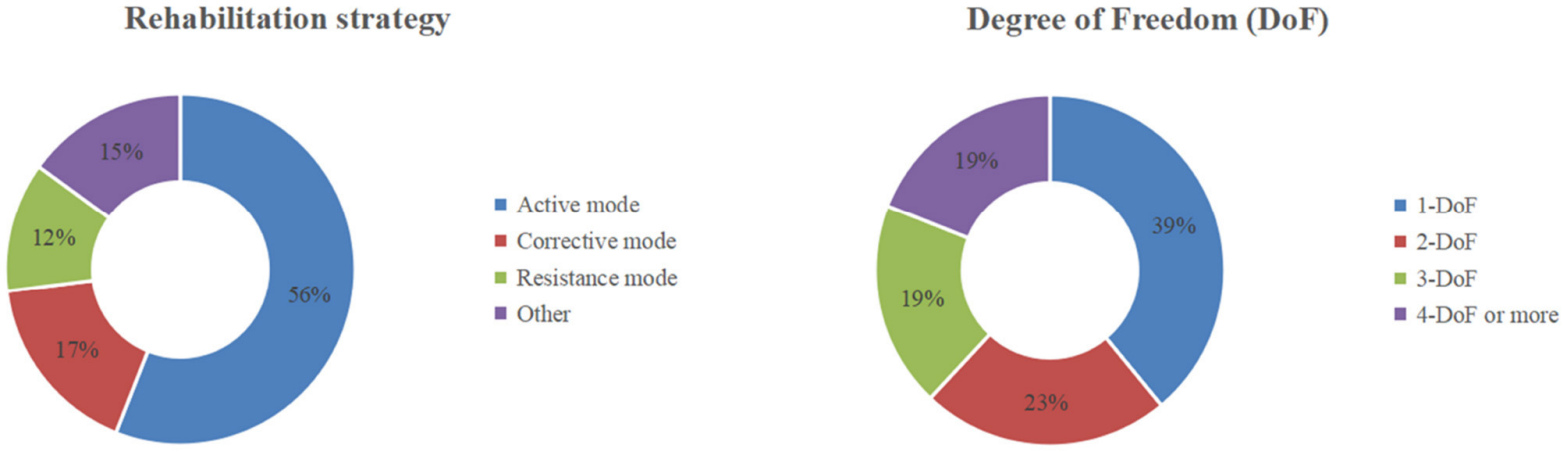
The distribution of the operating modes of exoskeletons in terms of rehabilitation strategy and total DoFs (Vélez-Guerrero et al., 2021).

According to the control modes and the number of degrees of freedom (DoFs), the aspects linked to the robot exoskeleton function that have been documented in the literature might be grouped. The distribution of working modes can be seen in Figure 4.

#### 2.3.1. Rehabilitation strategy

**Figure 5:**
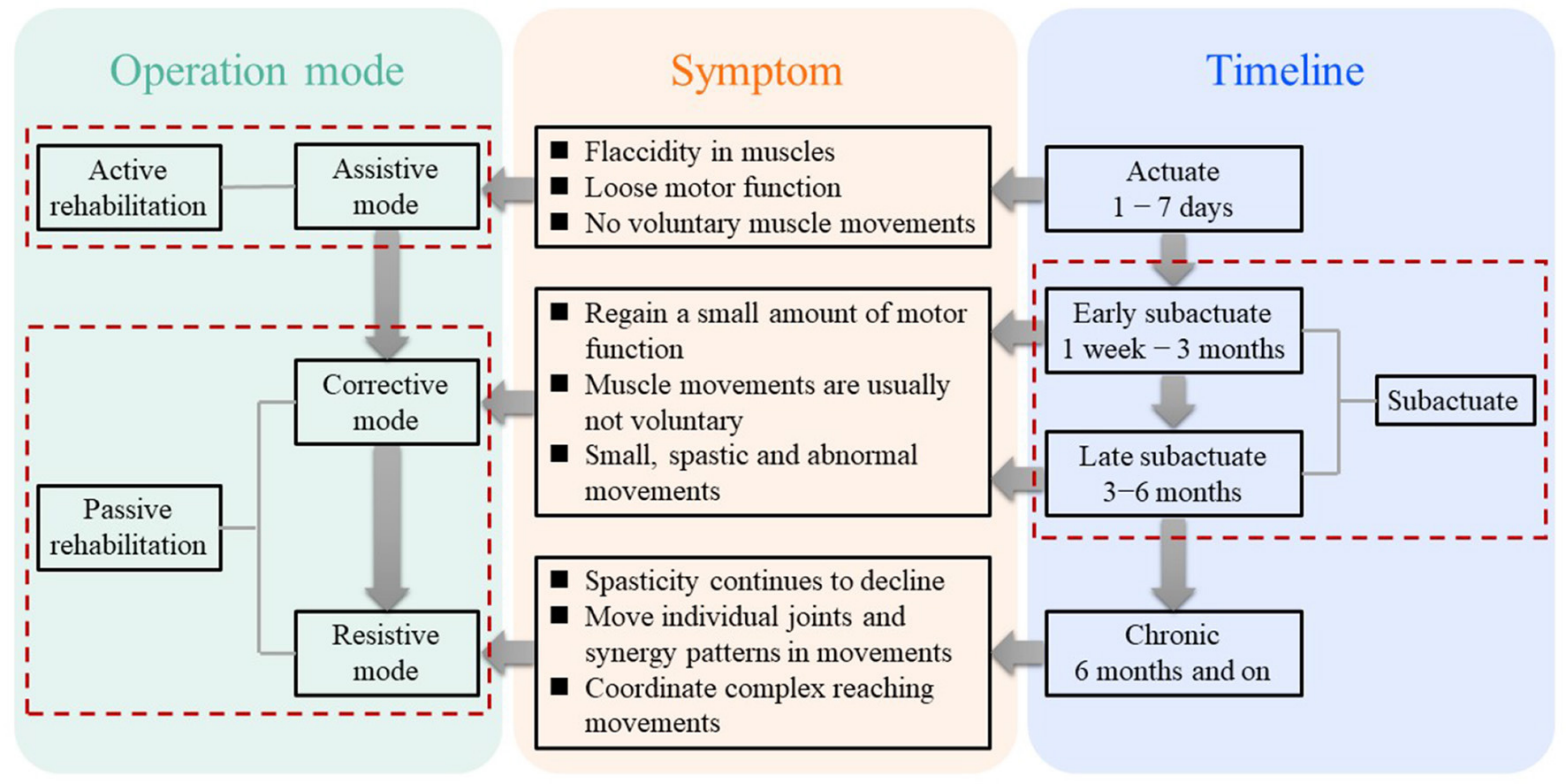
Three timeline phases after a stroke, corresponding symptom for each phase, and corresponding operation mode for each symptom.

Numerous researchers have looked into the physiological changes that occur in stroke victims. Signe Brunnstrom developed the “six stages of recovery” theory based on the various motor function recovery features, and it serves as a crucial theoretical guide for creating the rehabilitation strategy of a rehabilitation robot (Maceira-Elvira et al., 2019). Figure 5 depicts the three stages of the patients' post stroke stages based on Signe Brunnstrom's theory, the symptoms and the operation modes of the exoskeleton associated with each stage.

**Figure 6:**
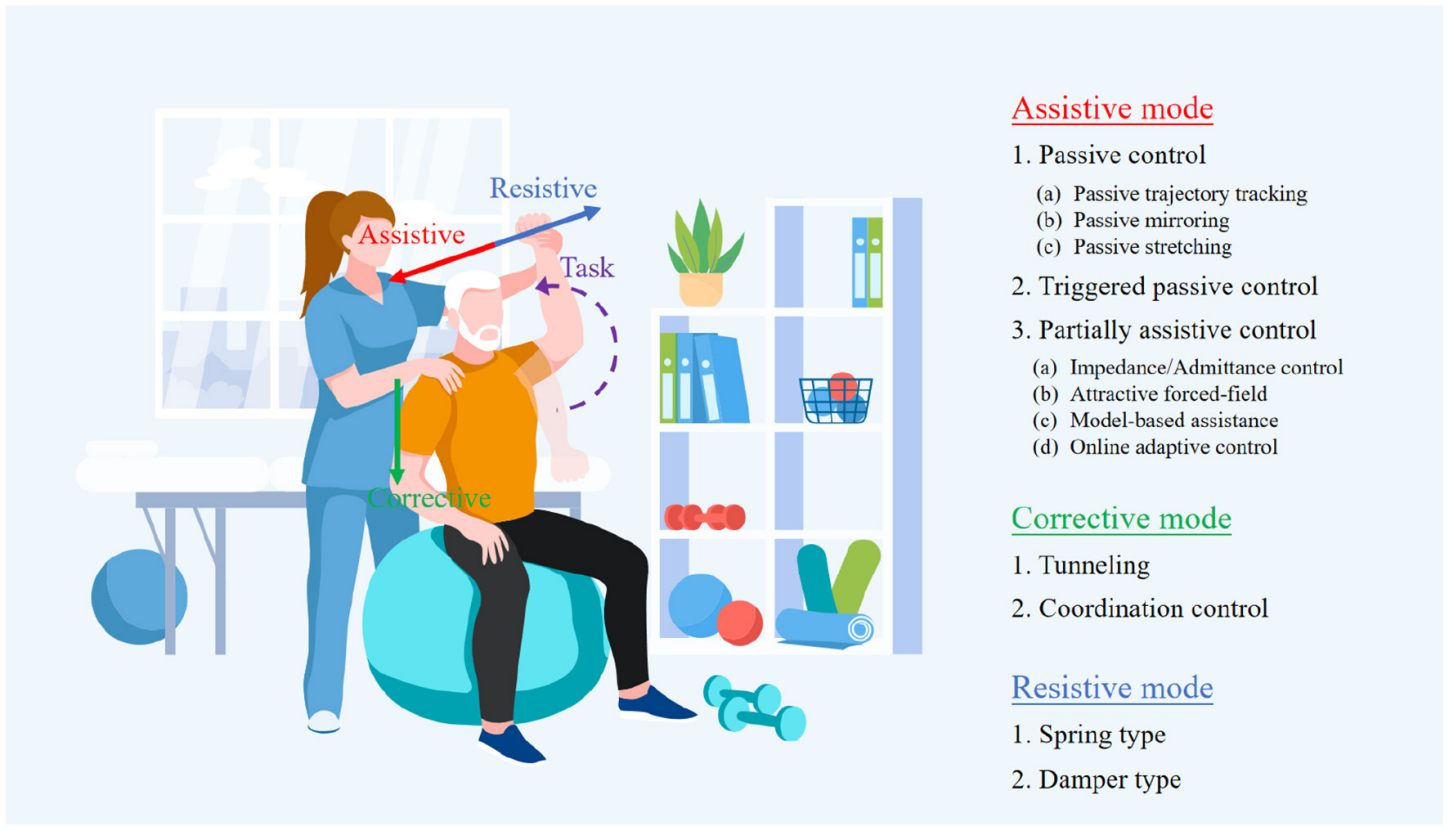
Operation modes of exoskeleton for people with different levels of strokes.

In the rehabilitation of motor or neuromotor function, three control modes are usually considered: active mode, passive/corrective mode, and resistance mode, as shown in Figure 6. The active control mode provides all necessary motions to the limbs through the robot exoskeleton, accounting for 56% of the study. In the sample, 17% of the literature reported the use of corrective control mode, which refers to the use of an exoskeleton accompanied by limb movement, and the resistance mode opposes the movement of limbs, accounting for 12% of the literature.

##### 2.3.1.1. Assistive mode

Within the assistive mode, there are three types of operation modes: passive, triggered passive, and partially assistive. It is noted that these three different types are usually mixed in practice. In the early stages of recovery from a stroke, the individuals typically lose all fundamental movement skills in the impaired limbs. Therefore, in passive operation mode, the exoskeleton should provide both the force to support the weight of the impaired limb and the force to perform the motor task without the individuals generating any force to perform movements. One feasible solution is to provide a controller with high feedback gains. However, the values of these gains must be carefully adjusted to prevent the exoskeleton from injuring the individuals because the large force for driving might cause muscle strains. In triggered passive mode, the movements of the exoskeleton are initiated by processing the cerebral signals from a device, such as a Brain-Computer Interface (BCI). This mode is suitable for individuals who are not likely to recover, for example, suffering from tetraplegia. Since the pure passive mode has a limited impact on neuroplasticity (Lynch et al., 2005), its primary function is to boost the confidence of the individuals in re-obtain the capability of voluntary movements. The partially assistive mode is often utilized when the patient has recovered just a little amount of motor function. In this mode, the patient and the exoskeleton may work together to govern movement, enabling the patient to express motions that are not constrained by their motor abilities. A typical and effective example is that Chowdhury et al. (2019) developed an exoskeleton with a partial assistive mode that determines rehabilitation based on the amount of fingertip force provided by patient. In detail, when the fingertip force exceeds a certain threshold, the device will allow the patient to accomplish motor tasks without assistance from the exoskeleton. The exoskeleton will, however, fully aid the patient in finishing the motor task when the fingertip force provided by patient is less than that threshold amount. This cutting-edge approach to rehabilitation is intended to encourage unrestricted engagement in therapeutic exercises, which is frequently thought to be advantageous for stroke patients' recovery.

##### 2.3.1.2. Corrective mode

The corrective mode, unlike the assistive mode, only provides supportive force when the subject is unable to perform the expected movements. With the supportive force, the impaired upper limb is forced by the exoskeleton to recover the desired inter-joint coordination. In detail, the main distinction between the two modes is that the assistive mode allows the exoskeleton to drive the impaired arm to the target position without the subject exerting any force, whereas the corrective mode is unable to accomplish this as the ideal corrective mode should not exert any force on the subjects when they have no effort in voluntary movement, but rather to reroute the subject's movement when it deviates until the upper limb trajectory is changed to conform to the expected trajectory. However, in practice, it is difficult to distinguish clearly between pure assistive mode and pure corrective mode because it is challenging to determine whether the exoskeleton is exerting any force to assist the individuals in performing the expected movement after they have regained some motor functions. One potential approach is that, in the assistive mode, the exoskeleton is set to drive the impaired arm to the stated expected position at the defining moment in time and finally arrive at the target position, whereas, this set is eliminated in the corrective mode, instead, an approximate movement path is provided rather than the precise movement trajectory planned in the assistive mode. Whenever the impaired arm movement deviates from this planned path, the exoskeleton corrects it along the orthogonal direction to return to the intended movement path until the target position is reached.

##### 2.3.1.3. Resistive mode

In resistive mode, the exoskeleton imparts resist individuals to accomplish the motor task. In this process, the exoskeleton continuously teaches them to perform corrective movements, and the individuals progressively regain their ability to move voluntarily and can adjust motions in response to external perturbations. The two primary categories of methods for implementing the demands of this mode are spring type and damper type. The spring type resists movement by returning the impaired limb to its starting position through an adjustable spring, whereas the damper type resists movement by an opposite force generated from a damper, and the value of that depends on the current velocity of the movement. However, it should be noted that although these require the patient to exert more force, it does help the individuals gradually recover to a level that is comparable to that of healthy people and enable them to accomplish precise motion tasks.

#### 2.3.2. The DoFs of the upper limb rehabilitation exoskeleton

The human arm can be simplified into seven degrees of freedom, namely, three degrees of freedom for the shoulder joint, one degree of freedom for the elbow, one degree of freedom for the forearm, and two degrees of freedom for the wrist. The degree of freedom of the rehabilitation robot is generally limited to these seven degrees of freedom. The articles presenting one degree of freedom are quite much, accounting for 39% of the review literature. These studies focus on the movement of different joints of the upper limb. Includes equipment operated on the wrist joint. Articles with two DoFs designs accounted for 23% of the reviewed literature. One special kind of the two DoFs models is very common: the shoulder joint and elbow joint model. The development of three degrees of freedom design accounted for 19% of the reviewed literature. Specifically, some researchers, such as Lei (2019), Wang et al. (2019), and Wendong et al. (2020), mainly establish degrees of freedom in the elbow joint, forearm rotation, and wrist joint. The main purpose of this exoskeleton is to provide rehabilitation treatment with a small, light, and high-performance equipment. In addition, the safety, ergonomics and rehabilitation theory of the device are also considered in their design. Articles with four DoFs or more also constituted 19%. Although the complexity of the rehabilitation motion that can be accomplished increases with the number of degrees of freedom, at the same time, an increase in degrees of freedom also makes it more difficult to design control systems, especially when the number of degrees of freedom exceeds the number of degrees of freedom required for the rehabilitation motion, which induces the redundancy problem, which has not yet been fully solved.

## 3. Challenges in upper-Limb exoskeleton development

Exoskeleton technology has advanced significantly in several areas, as was already indicated. But there are still a lot of difficult research problems that require attention. The following list includes a few design issues that are specifically connected to the design of exoskeletons.

### 3.1. Motion compatibility

The exoskeleton design should be kinematically compatible with the body parameters of the patients. How to correctly align the exoskeleton with the anatomical joint of the wearer is a challenging task. For instance, the exoskeleton mechanism supporting the shoulder joint and wrist joint should be a spherical joint in kinematics (Bai et al., 2019). The glenohumeral joint is usually called the ball and socket joint, which is formed between the humeral head and the joint in the glenoid cavity (Prinold et al., 2013). Most studies only considered the glenohumeral joint to simulate the mechanism of the three DoFs shoulder joint. However, as the human upper limb moves, the glenohumeral joint's instantaneous center of rotation (COR) shifts. As a result, it is imperative to account for the impact of the dynamic rotation center while simulating the external skeletal shoulder mechanism. While some studies have suggested a spherical system to mimic the shoulder and wrist's three degrees of freedom of motion, the majority of studies view the shoulder and wrist joints as ball and socket joints. The ideal way to develop a system that can adjust to the influence of the wrist and shoulder joints' instantaneous rotation centers can not be found yet by taking this aspect into account.

### 3.2. Discomfort

The English national quality standard states that stroke patients must be given a minimum of 45 min of each necessary therapy, at least 5 days a week. The vast majority of rehabilitation strategies for exoskeleton follow this standard, such as He et al. (2021), the participants underwent task-specific training involving 5-DoFs upper extremity movements in a three-dimensional environment with the aid of an exoskeleton for 45 min per day, 5 days per week, for 4 weeks (total of 20 sessions). In every session, the participants were required to complete two sets of exercises. Each set consisted of 5 min of passive training and 10 min of active training. It is very important to consider the exoskeleton's comfort because it has a significant impact on how stroke victims feel while wearing it for a long time. The comfort of wearable rehabilitation exoskeletons needs to be quantified, but there is no clear standard at present, which is one of the biggest problems. Ideally, the patient's physiological joints should be aligned with the corresponding joints in the exoskeleton. However, the alignment of the shoulder joint, wrist joint and thumb/finger joint is very difficult. Additionally, the exoskeleton connection on human limbs is frequently flimsy, which increases the possibility of sliding during a task between the exoskeleton and the body accessories. This deviation causes the exoskeleton and the human joints to dislocate, which will cause the human joints to produce unwanted response forces and torque.

### 3.3. Singularity problem of mechanical system

The exoskeleton's two joints' alignment with one another causes the singularity problem. The smooth manipulation of the exoskeleton by the human upper limbs is hampered by this issue. The exoskeleton loses one degree of freedom when this issue arises, and to correct the situation, a nearly endless amount of torque is needed (Islam et al., 2017). There are two ways to deal with this issue. One is to take into account potential scenarios when designing the control approach for an exoskeleton (Hsieh et al., 2017). According to Lee et al. (2014), the exoskeleton becomes unstable and may start to vibrate or collide with adjacent objects when it comes into contact with a singular configuration. They created a model-based force controller and a Jacobian pseudo-inverse in place of the inverse Jacobian using the singular value decomposition (SVD) method to overcome this issue. Additionally, SVD must be used in conjunction with the damped least square approach to tackle the problem because it has the potential to produce a huge value close to the unique configuration, which would make the system unstable. Another approach is to take into account various factors that may create singularity problems in the design of the exoskeleton by applying mechanical constraints and excluding them (Gull et al., 2020).

## 4. Discussion

The composition of an article on exoskeletons consists mainly of the physical properties of exoskeletons and their operation modes. Existing rehabilitation exoskeletons still have many problems in terms of movement coordination and comfort. Many exoskeletons do not take into account the complex motion patterns of the human shoulder and wrist joints, which can lead to problems in the coordination and comfort of the rehabilitation robot, such as the occurrence of motion asynchrony and the occurrence of undesirable extra moments in the rehabilitation movement. These problems are difficult to be solved in hard exoskeletons. Unfortunately, the vast majority of rehabilitation exoskeletons are hard exoskeletons. Although they have many advantages, their shortcomings such as lack of compliance, restrictive movement, and introduction of dislocation are difficult to eliminate, and it is difficult to wear them in public. Soft exoskeletons are a great option for joints with multiple degrees of flexibility, such as the shoulder, due to their compliance. The portability of the exoskeleton is also influenced by the materials used to make it, and the materials also determine the production cost of the exoskeleton. Generally speaking, mixed materials and plastic materials can find a suitable balance between strength and weight for the exoskeleton, and they can effectively reduce the cost of the exoskeleton.

Additionally, the operation mode of the exoskeleton directly determines the strength of its ability to rehabilitate patients. The more degrees of freedom a rehabilitation exoskeleton has, the greater the complexity and effectiveness of the rehabilitation movements it can perform. However, designing control strategies for multi-degree-of-freedom rehabilitation exoskeletons is challenging for developers. The existing rehabilitation exercise strategies of exoskeletons can basically cover the patient's need for rehabilitation exercise, but the question of how to generate the patient's rehabilitation movement trajectory is very important. On one hand, the exoskeleton must be provided with a feasible trajectory suitable for the human arm to successfully perform the required task, but on the other hand, taking into account the natural redundancy of the human arm, developers have to select one of many correct possibilities to perform the movement and complete the task. Furthermore, the ideal reference trajectory should be tailored to the patient's characteristics and take into account specific arm physical features and injuries such as paralysis, spasticity, and joint restrictions.

In summary, we believe that the biggest problem encountered in the development of portable exoskeletons at present is the problem of fitting complex joint motions. This problem can be solved by setting up special mechanisms in the rigid exoskeleton, such as a special linkage mechanism to adjust the rotation plane of the motor according to the patient's shoulder joint motion in order to fit the change of the patient's glenohumeral joint rotation plane. It is also possible to use flexible materials at complex joints or to use soft exoskeletons directly.

## 5. Conclusion

This paper focus on the mechanical design, control strategy, mode of actuation and power transmission, and exoskeleton design modeling based on the human upper-limb anatomy, new challenges in the research and development of this technology were also identified and discussed. This paper also examines the primary issues that are faced in the design of the current portable exoskeleton and offers a potential resolution.

## Author contributions

Conceptualization: JM and YZ. Methodology, resources, and software: YZ. Validation and formal analysis: HW. Investigation and writing—original draft preparation: YZ and HW. Data curation and writing—review and editing: MZ. Visualization: HW and MZ. Supervision and project administration: JM and MT. Funding acquisition: YZ and MT. All authors have read and agreed to the published version of the manuscript.
